# Milk-derived small extracellular vesicles: nanomaterials to promote bone formation

**DOI:** 10.1186/s12951-022-01580-w

**Published:** 2022-08-11

**Authors:** Ming Dong, Chun Shi, Xinxin Yu, Qian Yang, Saixuan Wu, Runyuan Liu, Tingjiao Liu, Lina Wang, Weidong Niu

**Affiliations:** 1 School of Stomatology, Dalian Medical University, Dalian, 116044 Liaoning China; 2 Department of Basic Science of Stomatology, Shanghai Stomatological Hospital, Fudan University, Shanghai, 200003 China; 3 Shanghai Key Laboratory of Craniomaxillofacial Development and Diseases, Fudan University, Shanghai, 200003 China

**Keywords:** Milk, Small Extracellular Vesicles, GJA1, AP3B1, Bone repair

## Abstract

Small extracellular vesicles (sEVs) are an important component in the paracrine pathway. They can be used as a substitute for seed cells and have shown good application prospects in promoting bone regeneration. Cow’s milk could be used as a source of sEVs with good biocompatibility and cost-effectiveness, with easy availability, low cost and low toxicity. This study focused on the role and mechanism of small extracellular vesicles derived from milk in bone repair. In order to explore the mechanism via which Milk-sEVs promote bone repair, we screened the differential gene GJA1 in Milk-sEV-treated osteoblasts through transcriptome chips, and verified the transcript AP3B1 of GJA1 through chromatin immunoprecipitation (CHIP). We have proved by in vivo and in vitro experiments that milk-derived sEVs (Milk-sEVs) increase the repair ability of bone tissue, and promote expression of the osteogenic gene GJA1 through the transcript AP3B1.

**Graphical abstract:**

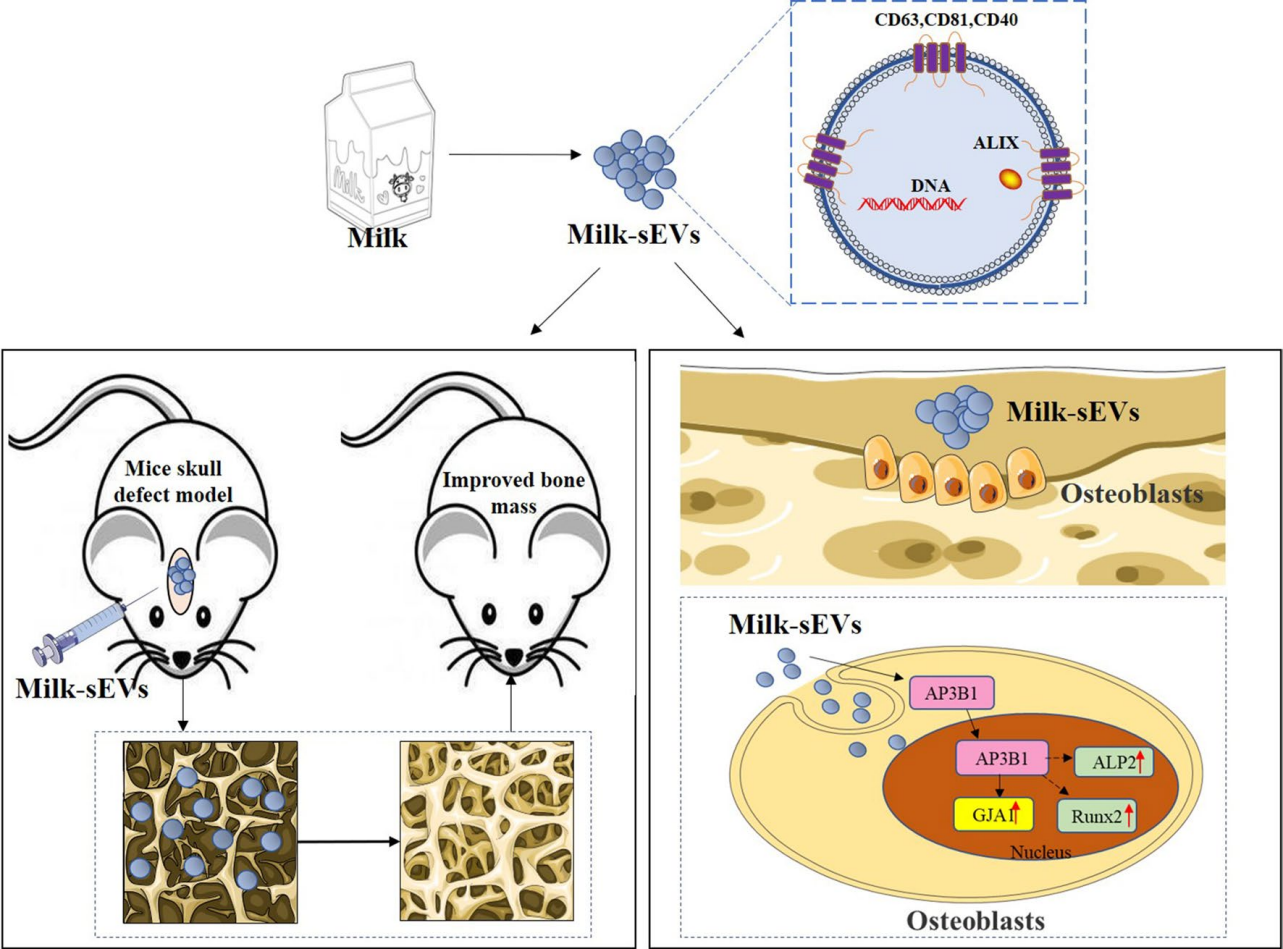

## Introduction

Extracellular Vesicles (EVs) are small membrane vesicles with a diameter of 20 nm–2 µm and a double-layered phospholipid structure [1, 2]. Extracellular vesicles can be divided into different subgroups, known as apoptotic bodies (ABs), microvesicles (MVs) and small extracellular vesicles (sEVs) [3]. sEVs can be generated by almost all types of cells under physiological and pathological conditions, and are widely present in body fluids such as blood, saliva, milk, urine, etc., where they are distributed as nano-scale EVs [4]. They carry a variety of intracellular genetic material, and act as important mediators, participating in mediating cell-to-cell communication, being involved in the regulation of various biological functions such as immune response, and participating in the occurrence and development of a variety of diseases [5–7]. Because of their unique source, structure and physiological function, sEVs are often used as an ideal natural endogenous nano-level medicine [8].

Bone defects in the oral cavity vary greatly, ranging from small intraosseous lesions caused by periodontal or peri-implant diseases to large jaw defects caused by trauma, tumor resection, or congenital defects [9, 10]. Alveolar bone repair depends on the dynamic balance between bone resorption and bone formation. In the past few decades, considerable effort has been devoted to exploring methods for repairing alveolar bone defects. The main method of clinical treatment for this problem is the transplantation of autologous or allogeneic bone, which is called “golden therapy” [11]. However, autologous bone grafts cannot provide enough bone for patients with larger defects, while allogeneic bone grafts cause more than 30% of patients to suffer from complications such as fractures and infections. Therefore, there is an urgent need to find new materials for the treatment of alveolar bone defects. Studies have shown that sEVs are safe, with good bone specificity and strong bone regeneration characteristics. Cui pointed out that osteoblast-derived sEVs increase the expression of miRNA related to osteoblast differentiation, inhibit Wnt signal transduction through Axin1, and promote the differentiation of HMSC cells into osteoblasts [12]. However, the high acquisition cost of sEVs makes it challenging to increase the yield of sEVs separation.

As a common food, cow’s milk is rich in a variety of proteins. sEVs derived from cow's milk have the advantages of low toxicity, high biocompatibility, physical and biological stability, good tolerance, and high cost-effectiveness [13, 14]. Milk could therefore be used to obtain a large number of sEVs. Studies have shown that the proteins in sEVs derived from bovine colostrum and mature milk (Milk-sEVs) have immunomodulatory effects, promoting the growth and proliferation of immune cells. Milk-sEVs have also been used as a nano-scale carrier in combination with small-molecule chemotherapeutic drugs to enhance the bioavailability of the drug and improve its efficacy and safety. Research by Agrawal showed that milk-derived sEVs can be used as a carrier for oral administration of the chemotherapy drug Paclitaxel (PAC). The ExoPAC exhibited lower immunotoxicity and systemic toxicity than traditional intravenous therapy [15]. Previous findings showed that milk sEVs accelerated osteoblastogenesis and reduced bone resorption [16, 17]. However, there is no report on the mechanism of how Milk-sEVs promote the proliferation of osteoblasts. In our study we successfully separated and identified Milk-sEVs by ultracentrifugation. We found that Milk-sEVs could promote the proliferation and differentiation of osteoblasts. In order to explore the mechanism via which Milk-sEVs promote bone repair, we screened the differential gene GJA1 in Milk-sEV-treated osteoblasts through transcriptome chips, and verified the transcript AP3B1 of GJA1 through CHIP. Through the above research, we hope to provide laboratory evidence for the clinical application of Milk-sEVs in alveolar bone repair.

## Materials and methods

### Separation of Milk-sEVs

sEVs were separated from fresh milk by ultra-high-speed centrifugation. Centrifugation was performed at 13,000×*g* for 30 min, followed by 100,000×*g* for 120 min, then the middle whey filter was collected, centrifuged at 130,000×*g* for 90 min, then 100,000×*g* for 120 min, and after filtration, centrifuged at 100,000×*g* for 60 min, and the Milk-sEVs suspension was collected.

### Western blotting

The protein concentration of each sample was measured using a QuantiPro BCA Assay Kit (KeyGen Biotech, Shanghai, China). The membranes were then incubated overnight at 4 ºC with specific anti-CD63 (diluted 1: 200; Abcam, Cambridge, MA, USA), anti-CD81 (diluted 1: 500; Abcam), anti-CD40 (diluted 1: 1000; Bioss Antibodies, Woburn, MA, USA), anti-ALIX (diluted 1: 1000; Abbexa Ltd., Cambridge, UK), anti-RUNX2 (diluted 1: 500, SAB, USA), anti-BMP-2 (diluted 1: 500, Bioworld Technology, St Louis Park, MN, USA USA), anti-ALP (diluted 1: 1000, Abcam), anti-GJA1 (diluted 1: 1000, Bioworld), anti-AP3B1 (diluted 1: 300, Proteintech, Rosemont, IL, USA) and anti-GAPDH (diluted 1: 5000, Bioworld). Incubation with the secondary antibody (diluted 1:500, ABclonal, Woburn, MA, USA) lasted 1 h. The ECL luminescent solution was configured to collect the blotting results with a Bio-Rad gel imaging system (Bio-Rad, Hercules, CA, USA), and the results were analyzed using Image Lab software.

### Real-time PCR

Total cell RNA was separated using Trizol, and the corresponding cDNA template was generated according to the reaction conditions of the reverse transcription kit (Takara Bio Inc., Shiga, Japan). The primers were designed and synthesized by Sangon Biotech (Shanghai, China). The primer sequences were AP3B1: Forward: 5'-GCCTTCCAGCCAAGATAACGT-3', Reverse: 5'-CGCAGCAGAACAGAACCAATC-3'; USF2: Forward: 5'-CTGTCCAAGGCCTTGCGATTAC-3', Reverse: 5'-TCGAAGCAGGGCATTCTCAT-3'; GJA1: Forward: 5'-CAGCGCAGAGCAAAATCGA-3', Reverse: 5'-R-GGTCGCTGTCCACGATAGC-3'; ALP: Forward: 5'-TGAATCGGAACAACCTGACTGA-3', Reverse: 5'-R-GAGCCTGCTTGGCCTTACC-3' and GAPDH: Forward: 5'- GTATCGGACGCCTGGTTA-3', Reverse: 5'- CATTTGATGTTAGCGGGAT-3'.

### Establishment of a mice skull defect model

Twelve 8-week-old male C57BL/6 mice were purchased from the Laboratory Animal Center, Dalian Medical University. The experimental protocol was approved by the Institutional Review Board of the School of Stomatology, Dalian Medical University (2021006). 1.2 μg Milk-sEVs were added to 100 ul GelMA (EngineeringForLife, China) to prepare a GelMA-Milk-sEVs composite material with a diameter of about 6.5 mm. After anesthesia (3% sodium pentobarbital, 0.8 mL/kg), the mice scalp was cut to strip away the periosteum, and the skull was drilled without pressure. A skull defect with a diameter of 1.5 mm was created and then the mice were randomly divided into three groups: a control group, a GelMA-PBS group and a GelMA-Milk-sEVs group. The control group was sutured directly without any treatment; in the GelMA-PBS group, GelMA-PBS hydrogel composite was placed in the defect and sutured; in the GelMA-Milk-sEVs group, GelMA-Milk-sEVs hydrogel composite was placed in the defect and sutured.

### Short interfering RNA (siRNA) knockdown experiments

MC3T3-E1 cells were seeded in six-well plates. For the knockdown experiments, siRNA targeting the AP3B1 (si-AP3B1; 200 nmol/well) and USF2 (si-USF2; 200 nmol/well) and a negative control siRNA were purchased from GenePharma (Suzhou, China). MC3T3-E1 cells were transfected with the Xfect RNA Transfection Reagent (TaKaRa, Dalian, China).

### Transcriptome sequencing

A total of 1 μg RNA per sample was used as input material for the RNA sample preparations. Sequencing libraries were generated using a NEBNext^®^ Ultra™ RNA Library Prep Kit for Illumina® (New England Biolabs (NEB), Ipswich, MA, USA) following the manufacturer’s recommendations and index codes were added to attribute sequences to each sample. The clustering of the index-coded samples was performed on a cBot Cluster Generation System using TruSeq PE Cluster Kit v3-cBot-HS (Illumina) according to the manufacturer’s instructions. After cluster generation, the library preparations were sequenced on an Illumina Novaseq platform and 150 bp paired-end reads were generated.

### Luciferase reporter assay

Luciferase reporter assay was performed in 293 T cells. DNA fragments encoding mice GJA1 promoters were ligated into pEZX-PG04.1 (GeneCopoeia Inc., Rockville, MD, USA) GJA1 promoter-luciferase reporter systems. Cells were then transfected in triplicate with one of the four vectors (GJA1-pomoter, Con-GJA1, Over-AP3B1, and Con-AP3B1). Gaussia luciferase (GLuc) activity and alkaline phosphatase activity were assayed after 48 h of transfection using a Secrete-Pair™ Dual Luminescence Assay Kit (GeneCopoeia) according to the manufacturer's instructions.

### Statistical analysis

The results were graphically depicted as the mean ± standard deviation (SD). Two-tailed t-test and one-way ANOVA were performed (SPSS 13.0 for Windows, SPSS, Chicago, IL, USA) to detect statistically significant differences. P value < 0.05 was considered statistically significant.

## Results

### Isolation and identification of Milk-sEVs

**Fig. 1:**
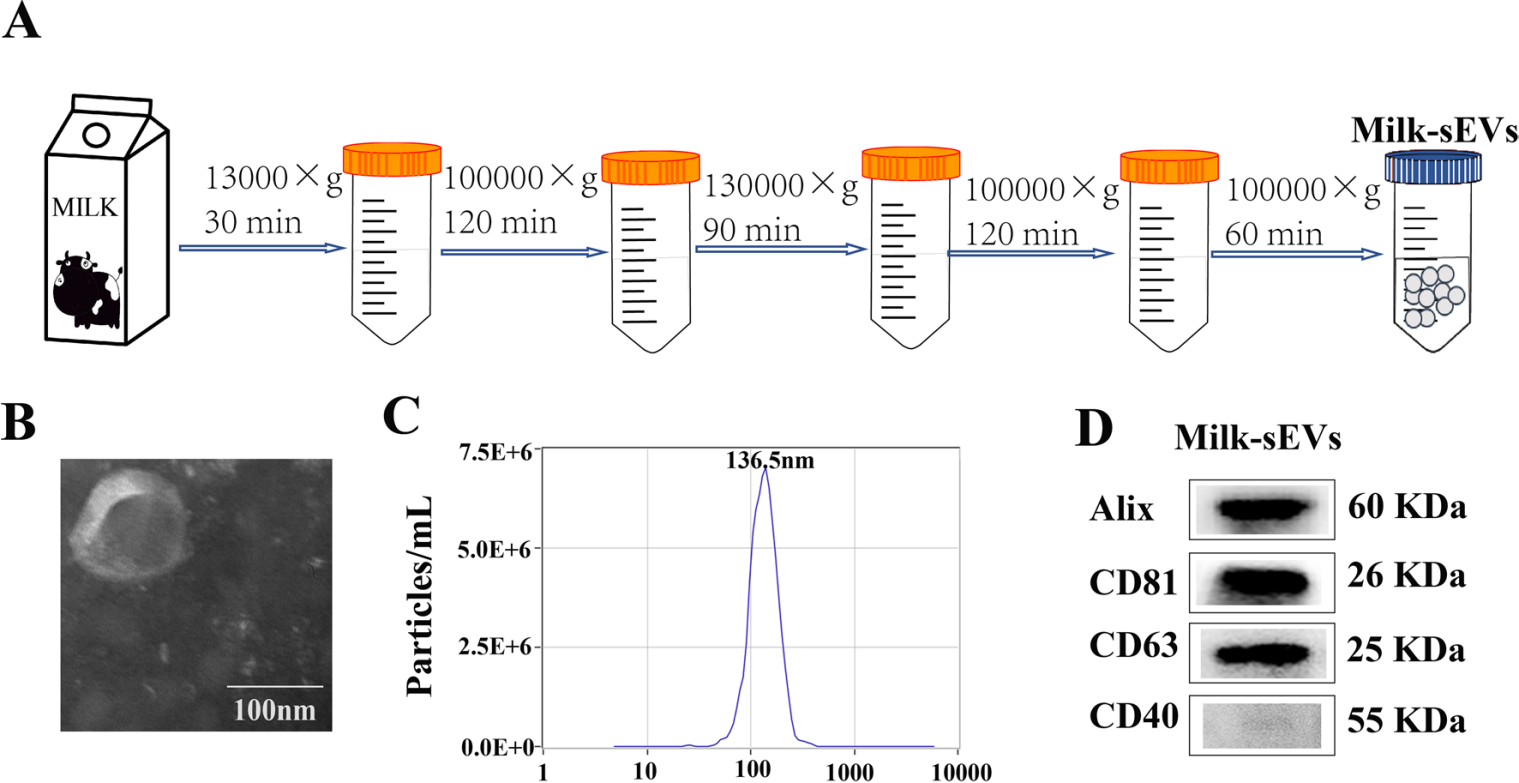
Isolation and identification of Milk-sEVs. **A** Schematic showing the extraction of Milk-sEVs by differential centrifugation; **B** observation of the structure of Milk-sEVs under TEM (× 100,000); **C** NTA detection of the median particle size of Milk-sEVs; **D** western blotting was used to analyze protein expression of the sEVs markers CD63, CD81, and ALIX and the microcapsule surface marker CD40 in different batches of Milk-sEVs

Milk-sEVs were successfully separated from fresh milk by ultra-high-speed centrifugation, and the extraction process is shown in Fig. 1A. The results of TEM showed that Milk-sEVs were relatively regular in shape, mostly circular or elliptical, with a typical double-layer membrane structure (Fig. 1B). NTA analysis showed that the median particle size of Milk-sEVs was 136.5 nm, and the particle concentration was 7.5E + 6/mL (Fig. 1C). The positive markers CD63, CD81, and Alix were expressed in Milk-sEVs, while the negative marker CD40 was not expressed in Milk-sEVs (Fig. 1-D). These results proved that we had successfully extracted Milk-sEVs.

### Milk-sEVs promoted the proliferation and differentiation of osteoblasts

**Fig. 2:**
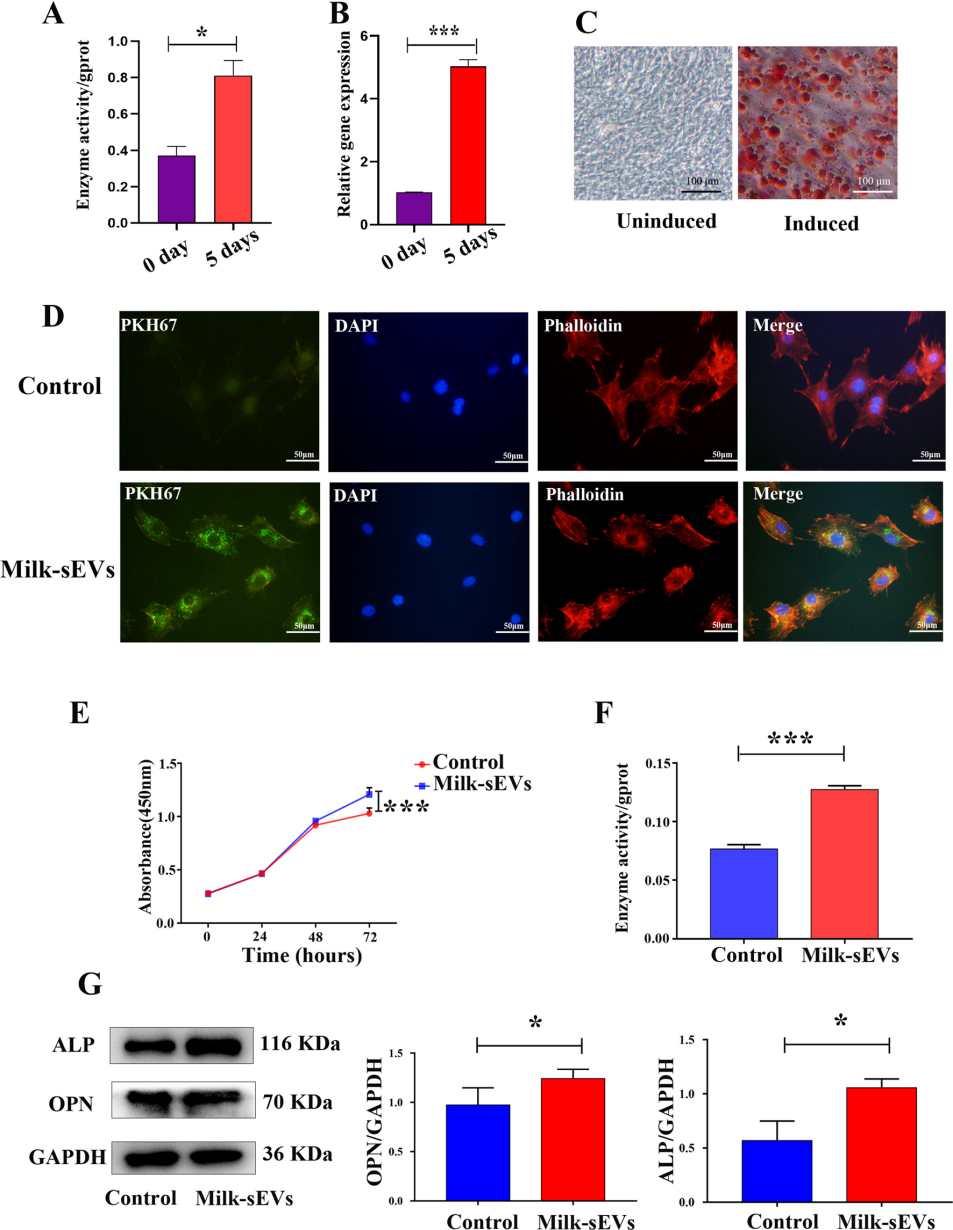
Milk-sEVs promoted the proliferation and differentiation of osteoblasts. **A** ALP assay results showing the ALP activity of MC3T3-E1 cells induced for 5 days; **B** Real-time qPCR results showing the relative expression level of ALP mRNA; **C** Alizarin red results after 21 days of cell induction; **D** fluorescence microscopy showing Milk-sEVs labeled with PKH67 fluorescence (scale bar = 50 μm); **E** CCK8 detection of proliferative ability; **F** ALP assay showing the expression level of ALP; **G** western blot showing expression of the osteogenic marker proteins ALP and OPN. The statistical results are presented as means ± SD, *P-value < 0.05, ***P-value < 0.001

ALP assay results showed that the ALP activity of MC3T3-E1 cells induced for 5 days was significantly higher than that of uninduced cells (P < 0.05) (Fig. 2A). Real-time qPCR results showed that the relative expression level of ALP mRNA in MC3T3-E1 cells induced for 5 days was significantly higher than that in uninduced cells (P < 0.05) (Fig. 2B). Alizarin red staining results showed that after inducing MC3T3-E1 cells for 21 days, a large number of dark red calcified nodules were seen on the cell surface (Fig. 2C). The above results proved the successful induction of osteoblasts. Control group (0 µg/ml Milk-sEVs) and Milk-sEVs group (20 µg/ml Milk-sEVs) were incubated with MC3T3-E1 cells. The Milk-sEVs were repetitively administered every 24 h. Observation under a fluorescence microscope showed that Milk-sEVs labeled with PKH67 fluorescence could be taken up by MC3T3-E1 cells after 24 h and distributed in the cytoplasm. The PKH67 fluorescence label of the control group was negative, as shown in Fig. 2D. The results of CCK-8 assay showed that the cell proliferation activity of the Milk-sEVs group at 72 h was significantly higher than that of the control group (P < 0.05) (Fig. 2E). The result of ALP assay showed that the expression level of ALP in the Milk-sEVs treated group was higher than that in the control group (P < 0.05), as shown in Fig. 2F. Western blot results showed that the protein expression levels of the osteogenic factors ALP and OPN in the Milk-sEVs-treated group were significantly higher than those in the control groups (P < 0.05) (Fig. 2G).

### Milk-sEVs promoted bone repair in mice skull defect model

**Fig. 3:**
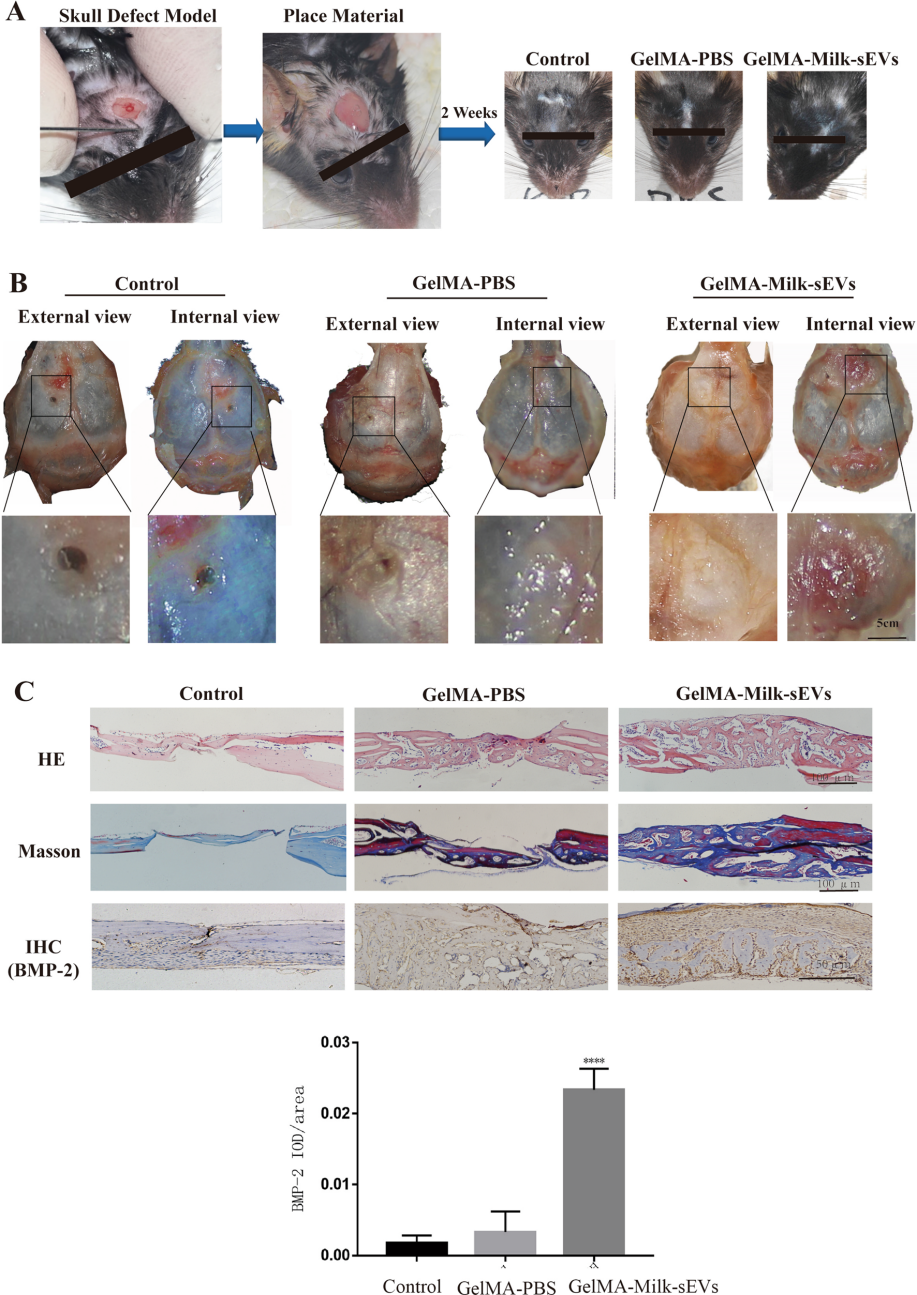
Milk-sEVs promoted bone repair in a mice skull defect model. **A** Schematic diagram of the mice skull defect model; **B** external and internal views of the skull defect model; **C** the result of HE staining (scale bar = 200 μm); the results of Masson staining (scale bar = 200 μm); the results of IHC staining; (n = 4/group) (scale bar = 100 μm). The statistical results are presented as means ± SD, ***P-value < 0.001

Two weeks after the operation, observation of wound healing in the mice defect model showed that the three groups healed well without any instances of infection, scab, ulceration or swelling, as shown in Fig. 3A. External and internal views of the skull defect model are shown in Fig. 3B. The control group did not exhibit any obvious bone tissue repair. In the GelMA-PBS group, the defect was closed in the internal view, and the surrounding fibrous tissue had proliferated, so that a small part of the defect was closed in the external view; in the GelMA-Milk-sEVs group, the internal view was filled with bone tissue, and the external view was completely closed, but a depression remained.

HE staining results showed that the size of the defect in the control group did not change, no new bone was formed in the center of the defect, and no new bone was formed under the periosteum or on the dura. In the GelMA-PBS group, there was a small amount of new trabecular bone formation in the center of the defect. New bone trabeculae were visible on the dura mater around the defect, while osteoblasts were seen in the bone trabeculae, but no new bone formation was observed under the periosteum; In the GelMA-Milk-sEVs group, there was a large number of new bone trabeculae within the defect, the defect was closed, a large number of new bone trabeculae were observed on the dura mater around the defect, new bone was formed in the bone marrow cavity, and there were a large number of osteoblasts. The results of Masson staining showed that no new bone was formed in the defect of the control group; in the GelMA-PBS group, a small amount of new bone tissue was stained blue at the defect site, and new bone trabeculae on the dural surface of the bone tissue around the defect were blue; in the GelMA-Milk-sEVs group, a large number of new blue-stained bone trabeculae were observed at the defect site, new blue-stained bone trabeculae were observed on the dural surface, and a small amount of mature bone tissue in the new bone was red. The results of IHC staining showed that there was a low level of expression of the bone formation marker protein BMP-2 in the defect site in the control group. The GelMA-PBS group also only had low expression of BMP-2 in the bone marrow cavity of the new bone tissue at the defect, while in the GelMA-Milk-sEVs group, osteoblasts on the surface of the new bone trabeculae in the defect and bone cells in the bone lacunae expressed high levels of BMP-2. The results of the control group and the GelMA-PBS group were not significantly different (P > 0.05), but the results of the GelMA-Milk-sEVs group were significantly different from both the control group and the GelMA-PBS group (P < 0.05).

### Transcriptome analysis of Milk-sEVs on MC3T3-E1 cells

**Fig. 4:**
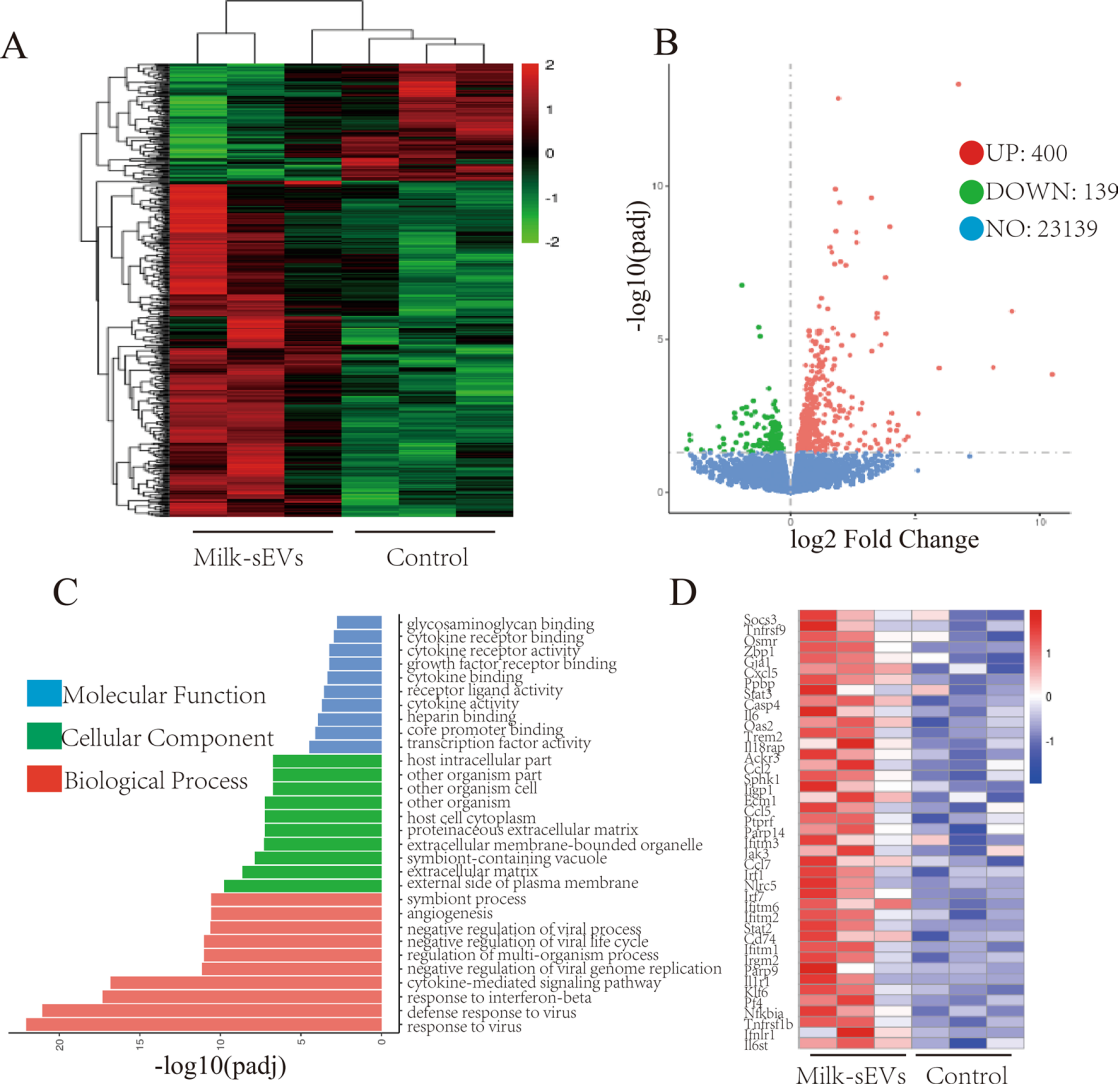
Transcriptome analysis of the effect of Milk-sEVs on MC3T3-E1 cells. **A** Heatmap of RNA sequencing data; **B** volcano plot of the differentially-expressed genes (DEGs); **C** GO analysis results showed that the top 10 DEGs were enriched at the BP, CC and MF levels; **D** RNA sequencing heat map analysis of the DEGs in the cytokine-mediated signaling pathway

The heat map shows the up/down range of the probe signal of the whole gene in the Milk-sEVs group and the control group. The deeper the red color, the greater the up-regulation of the gene, as shown in Fig. 4A. Compared with the control group, a total of 23,678 genes was identified in the Milk-sEVs group, of which 400 genes were up-regulated and 139 genes were down-regulated (P < 0.05). The volcano plot representing these differentially-expressed genes (DEGs) is shown in Fig. 4B. GO enrichment analysis revealed that the upregulated genes could be divided into biological process (BP), cellular component (CC) and molecular function (MF). The GO analysis results showed that the top 10 DEGs enriched at the BP, CC and MF levels are shown in Fig. 4C. Based on the above results, we found that the cytokine-mediated signaling pathway differed significantly in BP, so we have shown the DEGs in the cytokine-mediated signaling pathway (Fig. 4D). Through searching the literature, we found that the expression of GJA1 in the cytokine-mediated signaling pathway was significantly different, and GJA1 is also a marker of bone formation, so we used GJA1 as a key factor in our follow-up experiments.

### Milk-sEVs promoted osteoblast proliferation through AP3B1 transcribing GJA1

**Fig. 5:**
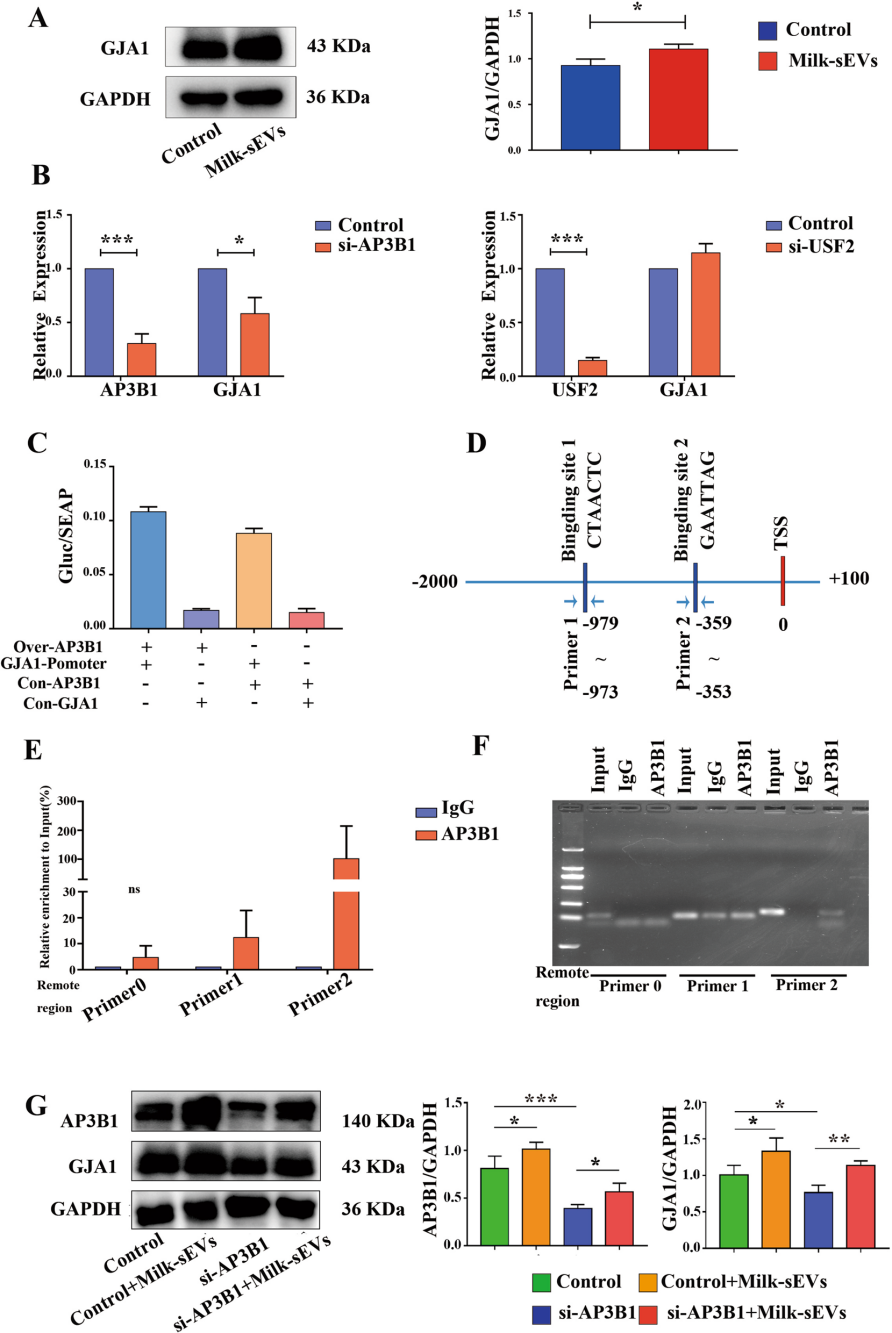
Milk-sEVs promoted osteoblast proliferation through AP3B1 transcription of GJA1. **A** The results of western blot; **B** the results of transfection of si-AP3B1 and si-USF2 into osteoblasts using RNA interference technology; **C** dual luciferase activity detected by co-transfection of Over-AP3B1 and plasmid GJA1-promoter, (+) indicated that Over-AP3B1 or GJA1-promoter was added, (−) indicates no addition; **D** we speculated that there were two specific binding sites for STAT5a on the GJA1 promoter; **E** the results of ChIP; **F** the results of agarose gel electrophoresis; **G** after addition of Milk-sEVs to si-AP3B1, western blotting showed that both AP3B1 and GJA1 expression increased. The statistical results are presented as means ± SD, ns: no significant difference. *P-value < 0.05, **P-value < 0.01, ***P-value < 0.001

Western blot results showed that after Milk-sEVs acted on osteoblasts, the expression of GJA1 was higher than that of the control group. This result was consistent with the results obtained by the transcriptome chip, as shown in Fig. 5A. Using bioinformatics, we predicted the two promoters of GJA1, AP3B1 and USF2. After transfecting si-AP3B1 and si-USF2 into osteoblasts using RNA interference technology, GJA1 expression decreased, while after transfection of si-USF2, expression of GJA1 increased (Fig. 5B), indicating that interference with AP3B1 affects the expression of GJA1. The GJA1 promoter sequence (−1000 impulse + 100) was cloned into the luciferase reporter vector pEZX-PG04.1 (GJA1-Promoter) and the transcription factor AP3B1 overexpressing plasmid (Over-AP3B1) was co-transfected to detect its luciferase activity. The results showed that the luciferase activity of the GJA1-Promoter + Over-AP3B1 group was significantly higher than that of GJA1-Promoter group. The luciferase reporter gene method confirmed that AP3B1 specifically binds to the GJA1-Promoter, as shown in Fig. 5C. The PROMO database was used to predict the specific binding site of AP3B1 on the GJA1 promoter. We speculated that there must be two specific binding sites for STAT5a on the GJA1 promoter, as shown in Fig. 5D. ChIP determined its actual binding site. Real-time qPCR based on immunopurified DNA fragments showed that both predicted binding sites of AP3B1 on Gja1 were larger than those of the control group, as shown in Fig. 5E. The PCR results of agarose gel electrophoresis also provided additional support for the ChIP detection results. The above results confirmed that AP3B1 directly binds to the two sites of the GJA1 promoter to regulate the expression of GJA1 (Fig. 5F). After we added Milk-sEVs to si-AP3B1, both AP3B1 and GJA1 expression increased, showing that Milk-sEVs up-regulated the expression of AP3B1, and AP3B1 combined with the binding site on the GAJ1 promoter to activate GJA1 transcription.

## Discussion

SEVs are widely present in plasma, urine, saliva, amniotic fluid, ascites and cerebrospinal fluid [18]. However, as a common food, cow's milk is currently the biological fluid containing sEVs. Hata isolated Milk-sEVs and proved that they contain mRNA and miRNA, and that miRNA could enter recipient cells through endocytosis, which is essential for cell-to-cell communication and material transfer [19]. Shu showed that Milk-sEVs had cross-species bioavailability, while animal experiments showed that Milk-sEVs were not toxic by intravenous injection or oral administration, and there were no abnormalities in blood parameters or inflammatory factor levels, which proved that Milk-sEVs had low toxicity and high biocompatibility [20]. Our group successfully isolated, extracted and identified Milk-sEVs. In vitro studies also confirmed that Milk-sEVs promoted the proliferation and differentiation of osteoblasts. Therefore, it was necessary for us to conduct in vivo studies to further verify their osteogenic effects.

In this experiment, GelMA hydrogel was used as a drug carrier and loaded with Milk-sEVs to achieve local sustained release. Zhao loaded paclitaxel onto the hydrogel and observed that paclitaxel was released in bursts on the first day then released slowly over the following week, and could still be detected after 4 weeks [21]. This confirmed that GelMA hydrogel could be used as a drug-controlled release system. The cured hydrogel had micropores and could be used as a drug carrier to slowly release drugs locally [22]. In order to increase the drug loading as much as possible, in this experiment, Milk-sEVs were lyophilized and re-formed into a suspension, then mixed with GelMA solution, and quickly solidified in a 96-well plate to prepare a hydrogel composite with a diameter of approximately 6.5 mm. The composite was placed on the missing part of the mice skull defect model, so that the Milk-sEVs were slowly released locally.

In vivo experiments showed that there was no new bone tissue in the defect of the control group, and no obvious BMP-2 expression was found. In the GelMA-PBS group, a small amount of trabecular bone was formed in the defect, and new bone trabeculae appeared on the dura surface of the bone tissue around the defect. The reason for this was that GelMA itself could act as a three-dimensional scaffold for cell growth to enhance bone formation ability. Studies have shown that GelMA enhances the adhesion of adipose-derived stem cells, increases their ALP activity and the mRNA expression level of osteogenic genes [23]. It has also been proved that it can be used as a carrier of adipose-derived stem cells to enhance bone formation in a mice skull defect model [24, 25]. Dong’s study also confirmed that GelMA has a certain osteogenic effect, which was similar to our results, but its osteogenic effect was limited [26]. Our IHC results showed only a small amount of BMP-2 expression. When the results were compared with the control, there was no significant difference between the groups. In the GelMA-Milk-sEVs group, a large amount of new bone tissue was formed at and around the defect. This result indicated that the GelMA-Milk-sEVs promoted bone remodeling of the skull. The results of IHC staining showed high expression of BMP-2 in this group, and statistical analysis showed that the results were significantly different from those of the other two groups. Pieters pointed out that Milk-sEVs have a certain immune function. They showed that these extracellular vesicles carried bioactive TGF-β, and that anti-TGF-β antibodies blocked Th17 differentiation [27]. TGF-β can increase ALP1 expression in preosteoblasts, which may be the reason why Milk-sEVs promoted bone formation [28]. Therefore, we considered that GelMA-Milk-sEVs enhanced the osteogenic differentiation at the defect site. Different from using β-TCP to load sEVs into the defect, GelMA was used as a drug delivery material in this study [29]. We solidified the GelMA solution combined with Milk-sEVs into a composite material with a diameter of 6.5 mm. The size exceeded the size of the defect (1.5 mm in diameter), which increased the concentration of Milk-sEVs in the area covered by the material. The area of new bone formation was not limited to the site of the defect, but instead new bone formation was also observed in the normal bone around the defect.

In order to study the mechanism of action of Milk-sEVs in bone repair, we screened out the DEGs after Milk-sEVs stimulation of osteoblasts using the transcriptome chip. Our results showed that cytokine-mediated signaling pathways were significantly different in BP. According to our literature survey, we found that GJA1, which is differentially expressed in cytokine-mediated signaling pathway, was also a marker of osteogenesis, so we used GJA1 as a key factor in our follow-up experiments. Studies had shown that GJA1 expression was up-regulated during osteogenic differentiation, while knockdown of GJA1 attenuated the osteogenic differentiation of BMSCs [30, 31]. Starting from the 9th day of differentiation of hBMSCs to osteoblasts, GJA1 expression exhibited a trend of up-regulation [32]. After knocking down GJA1, expression of the osteogenic-related factors Runx2, ALP, BSP, and OCN decreased [33]. Using bioinformatics, we predicted that the two promoters of GJA1 were AP3B1 and USF2. After transfecting si-AP3B1 and si-USF2 into osteoblasts using RNA interference technology, the results indicated that interference with AP3B1 would affect the expression of GJA1. We found that the transcription factor AP3B1 specifically binds to the GJA1 promoter and activates transcription of GJA1, while USF2 cannot bind to the AQP3 promoter and activate the transcription of USF2. In order to further determine the binding site of AP3B1 on the GJA1 promoter, according to the JASPAR, PROMO, and TRANSFAC database predictions, we speculated that there were two AP3B1 sites on the GJA1 promoter. The experimental results showed that AP3B1 specifically bound to the GJA1 promoter region. After addition of Milk-sEVs to si-AP3B1, both AP3B1 and GJA1 expression increased. The above results indicated that Milk-sEVs up-regulated the expression of GJA1 through AP3B1.

sEVs are a type of natural biomaterial with great application prospects, and many studies on sEVs are currently being carried out in the field of oral regeneration. However, the cost of obtaining sEVs derived from cells and serum is high, and the yield of donors is low. We need to find a more cost-effective source of sEVs. Due to the high availability, low cost, and cross-species tolerance of milk, we chose to isolate sEVs from milk, and study the effect and mechanism of promoting bone repair in both in vivo and in vitro experiments, hoping to provide new ideas for clinical treatment of bone destruction.

## Conclusion

In summary, bone metabolism is a complex process. If this dynamic equilibrium is disrupted, bone metabolic disease can easily ensue. In order to explore the mechanism of Milk-sEVs in bone formation, we verified through bioinformatics and chip experiments that Milk-sEVs up-regulated the expression of the osteogenic marker GJA1 through AP3B1. Through the above experiments, we hope to provide a certain reference basis for the clinical application of Milk-sEVs in the treatment of bone destruction-related diseases.

## Data Availability

All data and materials are available on request.

## Abbreviations

sEVs: Small extracellular vesicles
GJA1: Gap Junction Protein Alpha 1
CHIP: Chromatin immunoprecipitation
TEM: Transmission Electron Microscope
AP3B1: Adaptor Related Protein Complex 3 Subunit Beta 1
HMSC: Marrow stromal stem
ALP: Alkaline phosphatase
CCK-8: Cell Counting Kit-8
NTA: Nanoparticle Tracking Analysis
BMP-2: Bone Morphogenetic Protein 2
RUNX2: RUNX Family Transcription Factor 2
GelMA: Gelatin-methacryloyl
